# Sodium New Houttuyfonate Inhibits Cancer-Promoting *Fusobacterium nucleatum (Fn)* to Reduce Colorectal Cancer Progression

**DOI:** 10.3390/cancers14246111

**Published:** 2022-12-12

**Authors:** Fengjing Jia, Qun Yu, Ling Zhao, Yunhui Shen, Haidong Guo, Feng He

**Affiliations:** 1 Academy of Integrative Medicine, Shanghai University of Traditional Chinese Medicine, Shanghai 201203, China; 2 School of Pharmacy, Shanghai University of Traditional Chinese Medicine, Shanghai 201203, China

**Keywords:** colorectal cancer, *Fusobacterium nucleatum*, SNH, cancer treatment, antimicrobial therapy

## Abstract

**Simple Summary:**

Colorectal cancer (CRC) is the third most common malignancy and the second most deadly cancer worldwide. Recent studies have uncovered the close relationship between Gram-negative anaerobic bacterium *Fusobacterium nucleatum* (*Fn*), commonly found in the human oral cavity and gut, and CRC development. Currently, there is no specific antimicrobial therapy for CRC treatment. Due to the antibiotic allergy, side effects, and resistance of existing antibiotic therapy, a new generation of antimicrobial therapy targeting specific CRC-promoting bacteria is urgently needed. In this study, we were looking for herb medicines and found that sodium new houttuyfonate (SNH), derived from the plant Houttuynia cordata Thunb, showed potent antibacterial activity on *Fn* with little toxicity toward host cells. Importantly, SNH inhibited *Fn*-induced inflammation and CRC growth promoted by *Fn*. Our findings of SNH with potent anti-*Fn* activity are promising for CRC treatment and provide an important foundation for future antimicrobial therapy for clinical CRC treatment.

**Abstract:**

Colorectal cancer (CRC) is a major cause of morbidity and mortality worldwide. Recent studies showed that the common anaerobe *Fusobacterium nucleatum* (*Fn*) is closely associated with a higher risk for carcinogenesis, metastasis, and chemoresistance of CRC. However, there is no specific antimicrobial therapy for CRC treatment. Herbal medicine has a long history of treating diseases with remarkable effects and is attracting extensive attention. In this study, we tested six common phytochemicals for their antimicrobial activities against *Fn* and whether anti-*Fn* phytochemicals can modulate CRC development associated with *Fn*. Among these antimicrobials, we found that SNH showed the highest antimicrobial activity and little cytotoxicity toward cancer cells and normal cells in vitro and in vivo. Mechanistically, SNH may target membrane-associated FadA, leading to FadA oligomerization, membrane fragmentation and permeabilization. More importantly, SNH blocked the tumor-promoting activity of *Fn* and *Fn*-associated cancer-driven inflammation, thus improving the intestinal barrier damaged by *Fn*. SNH reduced *Fn* load in the CRC-cells-derived mice xenografts with *Fn* inoculation and significantly inhibited CRC progression. Our data suggest that SNH could be used for an antimicrobial therapy that inhibits *Fn* and cancer-driven inflammation of CRC. Our results provide an important foundation for future gut microbiota-targeted clinical treatment of CRC.

## 1. Introduction

Colorectal cancer (CRC) is the third most commonly diagnosed malignancy and the second leading cause of cancer death worldwide [1]. Based on the projection of aging, population growth, and human development, the global number of new CRC cases is predicted to continue to increase. The increase in CRC incidence is mainly attributed to elevated exposure to environmental risk factors, such as shifting lifestyle and diet toward westernization. The increasing incidence and mortality of CRC pose a growing global public health threat. Furthermore, a rising incidence of early-onset CRC is emerging. Currently, a majority of patients are diagnosed in the advanced stage. The prognosis of advanced CRC is very poor, and the 5-year survival rate is about 10% [2].

Chemotherapy alone or in combination is the primary choice for the treatment of CRC, but the prognosis is unsatisfactory, especially for patients with metastatic lesions. Advanced CRC often harbor several mutations, including KRAS, NRAS, and BRAF mutations, as well as microsatellite instability (MSI)/mismatch repair (MMR) status and other oncogenic pathway dysregulations, which confers resistance to first-line treatment of advanced CRC, including the targeted therapies, monoclonal antibodies targeting anti-epidermal growth factor receptor (EGFR) and vascular endothelial growth factor receptor (VEGFR). Despite great progress in targeted therapy and immunotherapy of CRC in recent years, few patients benefit from them [3]. FOLFOXIRI (fluorouracil, oxaliplatin, irinotecan, and leucovorin) with targeted therapy are recommended for first-line treatment of advanced CRC by the National Comprehensive Cancer Network [4]. This regimen has brought some benefits for the survival of patients with advanced CRC, but there are significant adverse drug reactions. The tolerability and adverse drug reactions are obstacles to the widespread application of the regimen. It remains to be solved to select the best advantage population to benefit from first-line treatment. Different therapy regimens should be formulated according to the physical condition, genetic status, and tumor burden of cancer patients. So far, there is no unified treatment that can treat every patient with equal effects. In addition, the majority of patients with stage III/IV CRC still lack effective drugs. Cancer drug development remains an urgent unmet clinical need for CRC patients.

Host microbial organisms in the gut or oral cavity have crucial roles in modulating cancer susceptibility and tumor progression. CRC is particularly in intimate contact with the gut microbiota, whose metabolites and inflammatory factors are linked to CRC initiation, progression, and metastasis. *Fusobacterium nucleatum* (*Fn*), the common anaerobe in the human oral cavity, causes a variety of opportunistic infections [5]. *Fusobacterium* has been detected for the first time in large-scale tissue samples from patients with CRC [6]. Live strains of *Fn* were isolated from human colorectal biopsy samples and from patient-derived xenograft tumor models. More importantly, recent studies have shown that the abundance of *Fn* is correlated to the progression of colorectal carcinogenesis, cancer metastasis, and chemoresistance [7,8,9]. In particular, *Fn* is enriched in CRC [9] and the enrichment of *Fn* is associated with poor survival. Furthermore, *Fn* promotes the resistance of CRC to chemotherapy [7]. Considering the tumor-promoting roles of *Fn* on the various aspects of CRC development, current drugs targeting *Fn* have only been used to treat *Fn*-associated inflammation, it is unknown whether drugs targeting *Fn*, particularly those with fewer side effects, can induce CRC regression alone or in combinatory treatment with first-line treatment of advanced CRC.

Herbal medicine, an important part of traditional medicine, has a long history of playing an important role in medical care. It is widely used and increasingly relevant in the world today [10]. World Health Organization (WHO) Global Centre for Traditional Medicine (GCTM) estimates that around 80% of the world’s population use and benefit from traditional medicine or herbal medicine. It is estimated that over 40% of pharmaceutical formulations are based on natural products and landmark drugs, including aspirin and artemisinin, originated from traditional medicine. Herbal medicine is widely recognized in its influential global medical compendium [11]. For example, artemisinin is a famous herbal medicine to be used to effectively treat malaria and was recognized in the 2015 Nobel Prize in Physiology or Medicine [12].

Herbal medicine has also been demonstrated to have different beneficial effects on cancers in various stages, from improving symptoms and quality of life, and preventing cancer recurrence, to extending the survival of cancer patients [13,14,15]. Clinical studies showed that long-term herbal medicine treatment was associated with prolonging the survival of patients with stage II/III CRC [16]. Herbal medicine huang qin ge gen tang enhanced the antitumor activity of 5-fluorouracil by regulating the E2F1/TS pathway with no appearance of obvious toxicity [17]. Herbal medicine is considered a powerful supplement for the treatment of CRC [18,19]. In addition, herbal medicine has played an important role in the treatment of bacterial infection, allergic disease, liver disease, and other diseases [20,21,22,23].

In this study, we first selected six common herbal chemicals (phytochemicals), including the flavonoids or phenylethanol glycosides icariin, baicalin methyl ester, acteoside, salidroside, and echinacoside, as well as sodium new houttuyfonate (SNH), which were reported to have antibacterial activities, and tested their antimicrobial activities against *Fn*. More importantly, we examined whether anti-*Fn* phytochemicals can modulate *Fn*-induced inflammation and tumor promotion. We found that, in these tested antimicrobial chemicals, SNH is the best anti-*Fn* compound. We further evaluated the effects of SNH on the growth of *Fn*-associated CRC and the underlying mechanism. SNH inhibited the tumor-promoting effect of *Fn* by directly disrupting the bacterial membrane. Our result suggests a relevant adjuvant therapy targeting microbials and microbe-associated inflammation could improve the outcome of CRC treatment, particularly, by inhibiting *Fn*.

## 2. Materials and Methods

### 2.1. Antimicrobial Activity of Herbal Chemicals

The minimum inhibitory concentration (MIC) of J-I and its halogenated derivatives against *Fn* (ATCC 25586) were determined as described [24]. The minimum bactericidal concentration (MBC) of the peptides was determined based on their MIC [24]. The details were shown in the Supplementary Materials.

### 2.2. The Antimicrobial Mechanism of SNH

To understand the mechanism of SNH, the detection of intracellular hydrogen peroxide, outer membrane (OM) permeability assay, flow cytometric analysis [25], laser scanning confocal microscopy, and molecular docking [26] were tested. The details were shown in the Supplementary Materials.

### 2.3. Cell Viability Assay

Murine colon cancer cell line MC38 was purchased from the Chinese Academy of Medical Sciences. Human CRC cell lines HCT116, HT29, and human colon epithelial cell line NCM460 were purchased from the Shanghai Institutes for Biological Sciences of the Chinese Academy of Sciences. All cells were cultured in Dulbecco’s modified Eagle medium (Gibco, USA) supplemented with 10% Fetal Bovine Serum (Gibco, Grand Island, NY, USA), penicillin (100 U/mL), and streptomycin (100 μg/mL) at 37 °C in a humidified chamber with 5% CO_2_. Cell viability was determined by cell counting kit-8 (CCK-8) assay (Dojindo, Kumamoto, Japan). Cells were seeded in 96-well plates at the density of 1 × 10^4^ cells per well and cultured overnight prior to SNH treatment. Different concentrations of SNH were added with an equal volume to the wells in 6 replicates in each group for 72 h, followed by the addition of 10 μL CCK-8 reagent and incubation at 37 °C for 4 h before OD_450nm_ measurement [27]. OD_450nm_ was measured using a microplate reader (Molecular Devices, Shanghai, China) to determine cell viability. The experiments were independently repeated three times.

### 2.4. Effect of SNH on Cell Proliferation Assay

HCT116 cells were seeded at a density of 1 × 10^4^ cells per well in a 24-well plate and cultured overnight, followed by incubation with *Fn* (multiplicity of infection [MOI] = 1000) in the presence of different concentrations of SNH or PBS [28,29]. Cell counts were measured at 24 h, 48 h, and 72 h using a Countstar cell counter (ALIT, Shanghai, China). Each experiment was independently repeated three times.

### 2.5. Effect of SNH on Murine Colorectal Cancer with Fn Colonization

All mouse experiments were carried out under the protocol PZSHUTCM220627053 approved by the Ethics Committee of the Shanghai University of Traditional Chinese Medicine. The murine CRC model with *Fn* colonization was performed as described [7,30]. NU/NU nude mice were purchased from the Shanghai Slac laboratory animals center. Human CRC cells HCT116 were collected by trypsin digestion and washing by PBS. 1 × 10^7^ cells/100 μL PBS were subcutaneously injected into the right axilla of 4-week-old male NU/NU nude mice to establish the CRC xenograft model. Nine days after HCT116 inoculation, *Fn* was injected into the tumor every 3 days for 18 days in five groups: (1) Saline (control group); (2) *Fn*; (3) *Fn* + low-dose SNH (LSNH, 37.5 mg/kg); (4) *Fn* + high-dose SNH (HSNH, 75 mg/kg); (5) *Fn* + MET (positive group). SNH or MET was administered by intragastric gavage once a day for 18 days. The width and length of CRC tumors were measured every 3 days. Tumor volume = (A × B^2^)/2, where A and B are the length and width in cm, respectively. After the last intragastric administration, the mice were sacrificed for the collection of tumors and colon tissues. Tumor weight was recorded, and tumor and colon tissues were fixed in 10% formalin and then embedded in paraffin. 5 μm tumor sections were stained with Ki-67 monoclonal antibody (1:200, Servicebio, Wuhan, China).

### 2.6. Quantification of Bacteria

RNA extraction, reverse transcription, and quantitative polymerase chain reaction (qPCR) were used to quantify *Fn* and gene expression as described previously [7,31]. The details were shown in the Supplementary Materials.

### 2.7. Assessment of Intestinal Permeability and the Expression of the Proinflammatory Cy Tokines

The effect of SNH on the expression of tight junction proteins Claudin-1 and Zonula occludens protein 1 (ZO-1) were analyzed by qPCR to quantify tight junction proteins. The effect of SNH on the expression of the proinflammatory cytokines tumor necrosis factor α (TNF-α) and interleukin-1β (IL-1β) in HCT116-engrafted mice with *Fn* inoculation was detected by qPCR. The details were shown in the Supplementary Materials.

### 2.8. Statistical Analyses

Graphpad prism 8.0.2 was used for statistical analysis and graphing. Statistical significance was determined using a one-way analysis of variance (ANOVA) with Dunnett’s post-hoc analysis. All data are shown as mean ± Standard Deviation (SD), and *p* values < 0.05 were considered statistically significant. * *p* < 0.05, ** *p* < 0.01, *** *p* < 0.001, **** *p* < 0.0001, compared with *Fn* group. # *p* < 0.05, ## *p* < 0.01, ### *p* < 0.001, #### *p* < 0.0001, compared with the NC group.

## 3. Result

### 3.1. SNH Exhibits Potent Antimicrobial Activity In Vitro

Minimum inhibitory concentration (MIC) is defined as the lowest concentration of an antimicrobial that inhibits the visible growth of microorganisms and minimum bactericidal concentration (MBC) is the lowest concentration of an antimicrobial that prevents the growth of microorganisms after subculture on media [32]. The MIC and MBC values reflect the antimicrobial activity of antimicrobials against microorganisms in vitro. In this study, we first tested the antibacterial activities against *Fn* of six common herbal chemicals with different structural moieties including SNH, icariin, baicalin methyl ester, acteoside, salidroside and echinacoside (Figure S1), which were reported to have antibacterial activities. The antibacterial activities of these herbal chemicals against *Fn* were evaluated based on their MIC and MBC values. To measure the antimicrobial activity of several herbal chemicals, the MIC assays were performed by a 2-fold serial dilution method. As shown in Table 1, the MIC of SNH is 200 μM, the lowest among the tested herbal compounds, while MIC of icariin, baicalin methyl ester, acteoside, salidroside and echinacoside were all higher than 320 μM (Figure S2). Not surprisingly, the MBC of SNH is 2000 μM (Table 1). The result showed that SNH has the best antibacterial activity against *Fn* among these tested herbal chemicals.

### 3.2. SNH Disrupts the Integrity of Cell Membranes of Fn

**Figure 1:**
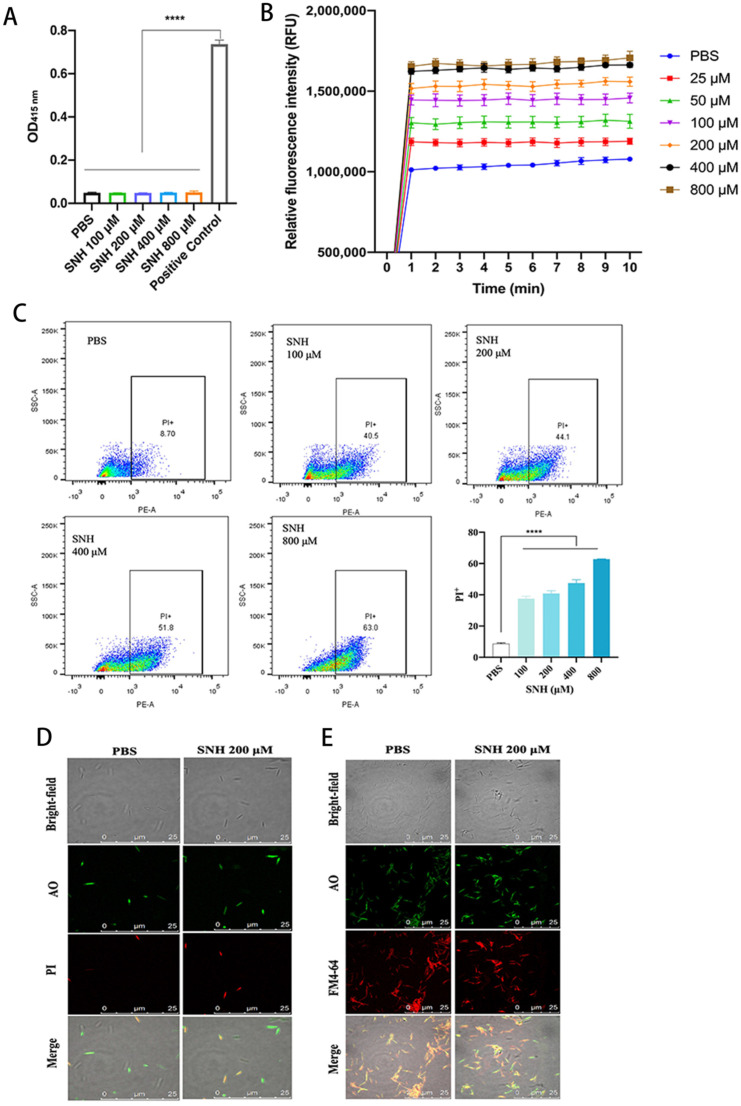
The effects of SNH on the level of reactive oxygen species and membrane integrity of *Fn*. (**A**) The effect of SNH on the intracellular hydrogen peroxide levels in *Fn.* (**B**) Dose–dependent outer membrane permeabilization of *Fn* induced by SNH. Time course of fluorescence of NPN bound to inner phospholipid membrane of *Fn* induced by different concentrations of SNH. PBS was used as a negative control. The experiment was performed independently three times. (**C**) Flow cytometry analysis of accessibility of propidium iodide (PI) to *Fn* genome in the absence or presence of different concentrations of SNH. PBS as a negative control. Bottom right shows the summary of PI^+^ *Fn* cells treated with increasing concentrations of SNH. The experiment was repeated three times, independently. All values were represented as mean ± SD. *p* Value < 0.05 was considered statistically significant. **** *p* < 0.0001, compared with the *Fn* group. (**D**) Confocal microscopic image of AO^+^ *Fn*, PI^+^ *Fn*, and AO^+^PI^+^ *Fn* in the absence or presence of SNH. (**E**) Confocal microscopic image of *Fn* co–stained with AO and FM4-64 in the absence or presence of SNH.

**Figure 2:**
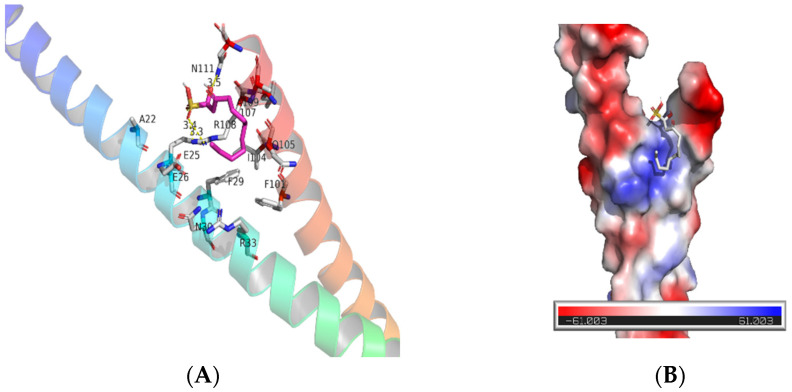
Molecular modeling of SNH binding to potential target FadA. (**A**) SNH interacts with the tail region of antiparallel α-helices of FadA (PDB: 3ETW) with the three potential hydrogen bonds shown in yellow dashed lines (distance labeled in Å). (**B**) Surface representation of FadA illustrating the SNH binding site. For clarity, of the interactions shown, only the protein segments that contain the groups of interest are shown. Hydrophobic, positively charged, and negatively charged surfaces are colored gray, blue, and red, respectively. FadA was shown in a rainbow cartoon from blue in N-terminus to Red in C-terminus. The residues around the binding groove of FadA and SNH are shown in sticks. N, O, and C atoms of side chains are colored blue, red and gray, respectively. SNH is in magenta. Molecular docking was carried out by Vina docking software, and the interactions between ligand and receptor were analyzed and presented by PyMOL.

To understand how SNH induces the inhibition and bactericidal effect of *Fn*, we first tested the level of intracellular hydrogen peroxide of *Fn*, which is a fairly common bacterial disruption mechanism for bacteria [33,34]. SNH did not alter the H_2_O_2_ formation in *Fn* at concentrations from 100 μM to 800 μM, at which *Fn* was already died (Figure 1A). We then explored the effect of SNH on the membrane integrity of *Fn*, and NPN was used to test the integrity of the outer membrane of bacteria. NPN is a fluorescent probe with a weak fluorescence signal in an aqueous environment, but it fluorescences strongly when bound to a hydrophobic phospholipid membrane of bacteria. A weak fluorescence signal of NPN was detected in the control group when NPN was co-incubated with *Fn* and PBS (Figure 1B). One minute after the addition of SNH (≥100 μM), the fluorescence signal quickly increased and reached a plateau. The maximal fluorescence intensity was further elevated with the increase in SNH concentration (Figure 1B). To confirm the effect of SNH on the inner membrane integrity of *Fn*, a DNA stain propidium Iodide (PI) uptake assay was performed and analyzed by flow cytometer. Extracellular PI is not cell membrane permeable. When the cell membrane is destroyed, PI can freely access DNA and fluoresces by binding to genomic DNA. SNH dose-dependently increased the fluorescence of PI in the presence of *Fn* (Figure 1C), indicating the loss of membrane integrity of *Fn* by SNH. To further confirm this, we used a confocal microscope to observe the localization of membrane-impermeable PI and membrane-permeable nucleic acid dye Acridine Orange (AO) in *Fn* with or without SNH. Consistent with the flow cytometry data, SNH treatment dose-dependently increased the PI binding to *Fn* DNA and resulted in the co-localization of PI and AO in *Fn*, indicating the loss of membrane integrity (Figure 1D). To further examine the effect of SNH on the membrane integrity, *Fn* were stained with lipophilic membrane colourant FM4–64 and AO in the presence or absence of SNH. When SNH was present, the FM4–64-stained membrane showed a discontinuous pattern with segregated puncta, compared with a more continuous pattern without SNH, suggesting disruption of membrane integrity induced by SNH (Figure 1E). To identify the potential molecular targets of SNH, we did a molecular docking analysis between SNH and structurally available membrane-associated proteins with oligomerization domains, including *Fusobacterium* adhesin A (FadA) [35]. We found that bacterial proteins FadA is a potential binding target of SNH with the binding free energy of −3.4 kcal/mol (Figure 2) and SNH binds to the tail regions of two antiparallel α-helices connected by an intervening 8-residue hairpin loop comprising the oligomerization motif of FadA, where the polar moiety of SNH forms three hydrogen bonds with FadA to facilitate the oligomerization, oxygen of hydroxy group of SNH interacts with the Gln 111 of α-helix 1, oxygens of sulfate interact with the Arg108 (Figure 2A). The surface presentation of SNH: FadA complex revealed that SNH sits in a partial hydrophobic groove formed by two α-helices whereas the polar moiety of SNH is facing the polar portion of the groove (Figure 2B). These data suggest that SNH disrupts the membrane by facilitating the oligomerization of membrane-associated FadA that penetrates and disrupts the membrane.

### 3.3. SNH Shows Little Cytotoxicity to Colon Cancer Cells and Colon Epithelial Cells

**Figure 3:**
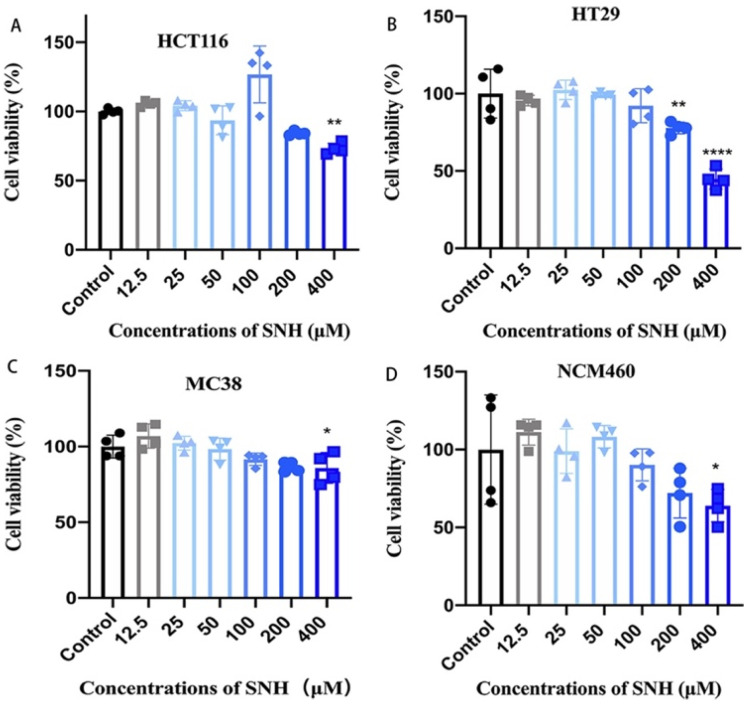
Cytotoxicity of SNH on different CRC cell lines and colon epithelial cells. Cell viability of human colon cancer cells HCT116 (**A**), HT29 (**B**), murine colon cancer cells MC38 (**C**), and human colon epithelial cells NCM460 (**D**) treated with various concentrations of SNH (12.5 μM–400 μM) for 72 h. Results were representative of three independent experiments performed in triplicates and were expressed as mean ± SD. *p* Value < 0.05 was considered statistically significant. * *p* < 0.05, ** *p* < 0.01, **** *p* < 0.0001, vs. Control (PBS vehicle group).

MIC and MBC of SNH to *Fn* is the significantly higher than those of metronidazole (MET) (Table 1), a common clinical antibacterial agent to treat various infectious diseases. Considering several severe side effects of MET clinically, we evaluated the cytotoxicity of SNH in both human/murine colon cancer cells (MC38, HCT116, and HT29 cells) and human colon epithelial cells (NCM460) by CCK-8 assay. Cells were treated with various concentrations of SNH for 72 h. When cells were treated with 200 μM of SNH, which is the *Fn*-MIC and showed potent antimicrobial activity, there is no toxicity observed for HCT116 and MC38 colon cancer cells and NCM460 cells (Figure 3). Although there was a decrease in cell survival for HT29, about 80% of HT29 cells were still alive (Figure 3). At the *Fn*-MBC concentration of 400 μM, all cells showed a decreased cell survival, but still higher than 60% of viability, except for the HT29, a little lower than 50% alive cells (Figure 3), indicating the significant cell tolerability of SNH.

### 3.4. SNH Inhibits the Tumor-Promoting Effect of Fn In Vitro

**Figure 4:**
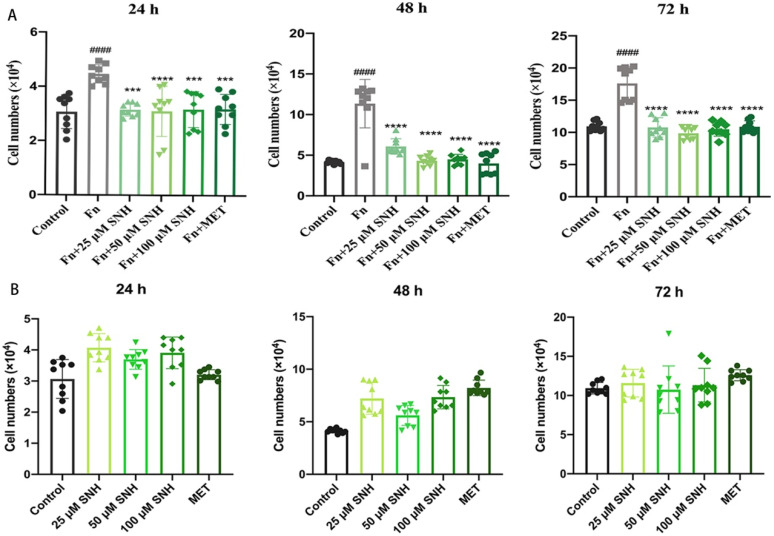
Effects of SNH on the proliferation of human colon cancer cells co-cultured with *Fn.* (**A**) Cell viability analysis of SNH on the proliferation of human colon cancer cells HCT116 co-cultured with *Fn* at the indicated time, metronidazole (MET) was used as a positive control. (**B**) Effects of SNH on the proliferation of HCT116 cells without *Fn* at 24 h, 48 h and 72 h, respectively. Each experiment was performed independently three times. All data were shown mean ± SD and *p*-value < 0.05 was considered statistically significant. *** *p* < 0.001, **** *p* < 0.0001, vs. *Fn* group. #### *p* < 0.0001, vs. Control.

To examine whether *Fn* promotes tumor growth and the effect of SNH on the proliferation of human colon cancer cells with *Fn*, we co-cultured HCT-116 cells with *Fn* in the presence or absence of SNH and analyzed cell viability at 24 h, 48 h, and 72 h. As shown in Figure 4A, compared with negative control, when the cells were incubated with PBS, co-culture with *Fn* increased the cell proliferation of HCT116 cells and the cell numbers were increased by about 30% and 100% at 24 h and 48 h. Even at 72 h, the cells still increased by about 70% when they reached the confluency. This suggests that *Fn* increases the proliferation of CRC cells, which likely explains the tumor-promoting effects of *Fn* observed in patients. When SNH was present, the cell proliferating effect of *Fn* was drastically inhibited with a cell number similar to that of the negative control. There were no significant differences at the doses of 25 μM, 50 μM, and 100 μM, and the inhibitory effect of SNH was the same as the positive control MET (Figure 4A). To rule out the possible effect of SNH alone on cell proliferation, HCT-116 cells in the absence of *Fn* were treated with the same concentration of SNH as when cells were co-cultured with *Fn* and SNH alone did not show any significant effect on the HCT16 proliferation (Figure 4B), consistent with its little cytotoxicity shown in Figure 3. These results indicate that SNH inhibited the tumor-promoting effect of *Fn* in vitro.

### 3.5. SNH Blocks the Growth of CRC Cell Line-Derived Xenograft Tumors Promoted by Fn Colonization

**Figure 5:**
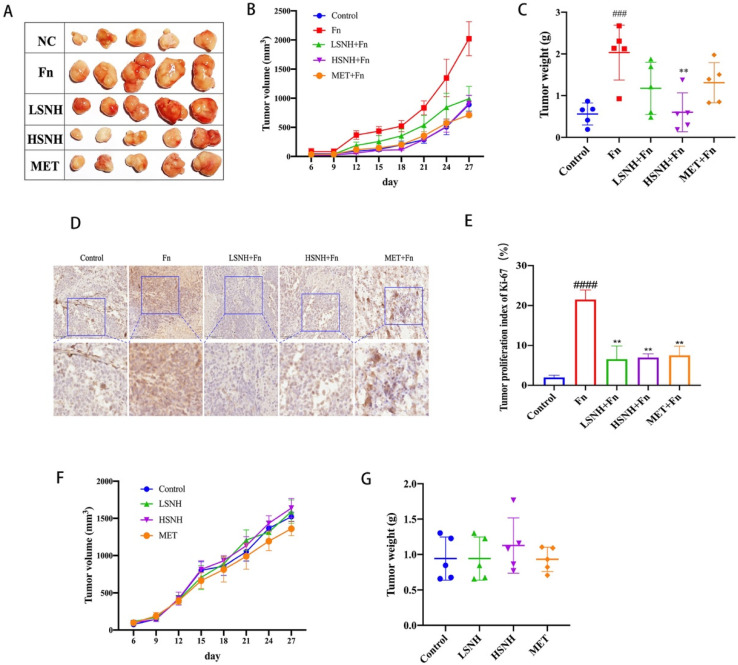
Effect of SNH on the growth of the CRC cells HCT116-xenograft tumors with intratumor colonization of *Fn*. (**A**–**C**), Gross image of tumors (**A**), tumor volume (**B**), and tumor weight (**C**) of tumors from the HCT116-engrafted mice inoculated with *Fn* in the presence of low-dose SNH (LSNH), high-dose SNH (HSNH), or the positive control drug MET. Time course effects of tumor volume were recorded in B. Endpoint of tumor appearance and tumor mass were shown in (**A**,**C**), respectively. (**D**) Immunohistochemistry of Ki-67^+^ cancer cells in the HCT116-tumor xenografts under different treatments as indicated. (**E**) Quantitation of Ki-67^+^ cancer cells in (**D**). (**F**,**G**) Tumor volume (**F**) and tumor weight (**G**) of HCT116 cells-xenografts in mice treated with LSNH and HSNH. All values were represented as mean ± SD, and *p* values < 0.05 were considered statistically significant. ** *p* < 0.01, vs. *Fn* group. ### *p* < 0.001, #### *p* < 0.0001, vs. Control.

To further examine the effects of SNH on the *Fn*-induced CRC tumor growth in vivo, we generated a human CRC cell line-derived mice xenograft model with intratumorally-colonized *Fn*. Immuno-deficient Nu/Nu mice were inoculated with human colorectal cancer cells HCT116 and after 9 days when tumor is formed, *Fn* was injected into the tumor. Tumor size and mass were recorded when SNH was intragastrically administrated daily until the mice were sacrificed. Consistent with the in vitro result, *Fn* inoculation increased the tumor volume and weight by more than 100% (Figure 5A–C). When low-dose SNH (LSNH, 37.5 mg/kg) or high-dose SNH (HSNH, 75 mg/kg) was administered, tumor-promoting effects of *Fn* were blocked. In addition, HSNH reduced the tumor volume and weight to a level similar to that of negative control, without *Fn* (Figure 5A–C). Tumors were then stained by proliferation marker Ki-67 to analyze their proliferation potential. *Fn* inoculation significantly promoted tumor proliferation of HCT116-xenografts, while lower proliferation was detected in the groups of treatment with LSNH or HSNH (Figure 5D,E). In addition, immunoblot of Caspase 3 from these tumor samples showed no significant changes in the total Caspase 3 and cleaved-Caspase 3 levels among different treatments (Figure S3), suggesting that the reduced tumor growth induced by SNH was due to the reduced tumor proliferation, rather than the increased tumor cell death. To rule out the effects of SNH on tumors directly, we treated HCT116-engrafted mice in the absence of *Fn* inoculation with SNH. At the same doses, SNH alone showed no effect on the tumor growth (Figure 5F,G). These results indicated that tumor-promoting effect of *Fn* was inhibited by SNH.

**Figure 6:**
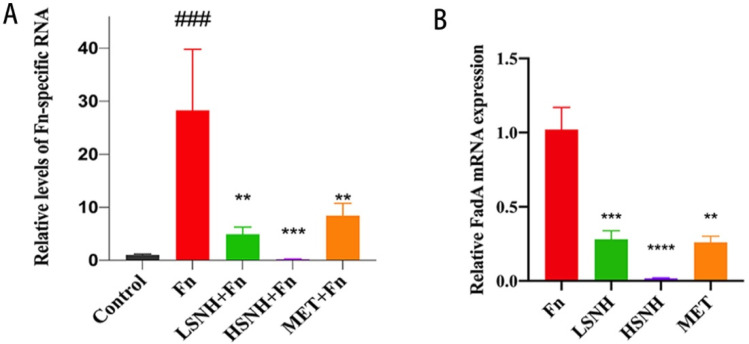
Effect of SNH on *Fn* in HCT116-xenograft tumors with intratumor colonization of *Fn*. (**A**) QPCR of *Fn*-specific RNA in the tumors of the mice engrafted with HCT116 cells with or without *Fn* colonization and SNH treatments. (**B**) QPCR of FadA RNA in the tumors of mice engrafted with HCT116 cells with *Fn* colonization and SNH treatment. All values were represented as mean ± SD, and *p* values < 0.05 were considered statistically significant. ** *p* < 0.01, *** *p* < 0.001, **** *p* < 0.0001, vs. *Fn* group. ### *p* < 0.001, vs. Control.

To examine whether SNH reduces tumor progression by directly inhibiting *Fn* proliferation in tumors, we quantitated *Fn* load within the tumor tissue by qPCR using *Fn*-specific primers. As a negative control, *Fn-*specific RNA was not found in the HCT116 xenograft tumors without *Fn* inoculation (Figure 6A). After *Fn* inoculation, *Fn-*specific RNA in the mice xenograft tumors was significantly increased, which is correlated with *Fn* load, and oral administrations of SNH at both low and high doses dramatically decreased the intratumor *Fn* load (Figure 6A). These results indicate that SNH reduced *Fn*-mediated growth of CRC tumors in vivo by directly inhibiting *Fn*. Consistently, the expression of the key *Fn* gene FadA within CRC xenografts was also reduced by SNH (Figure 6B).

**Figure 7:**
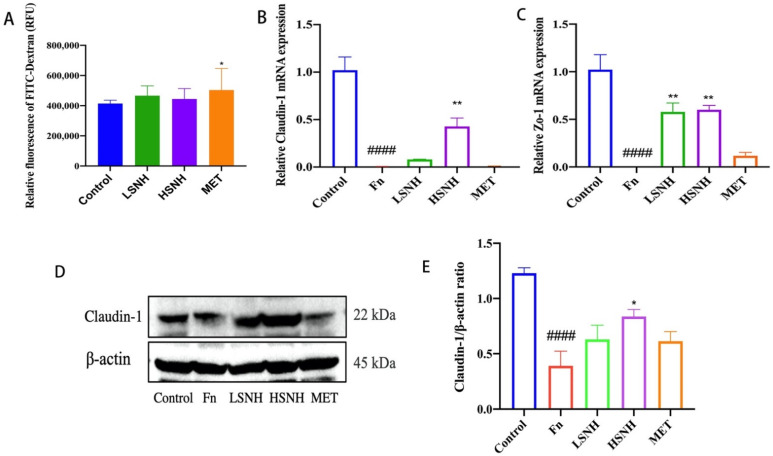
Effect of SNH on the integrity of intestinal barrier in HCT116-xenograft-bearing mice. (**A**) Effects of SNH and MET on the permeabilization of the intestinal barrier in the CRC cells-engrafted mice assessed by 4 kD FITC-Dextran permeability assay. Plasma was collected and measured the FITC-fluorescence intensity 4 h after the CRC-bearing mice were intragastrically gavaged with 4 kD FITC-Dextran. (**B**,**C**) qPCR analysis of the expression of genes encoding the tight junction proteins Claudin-1 (**B**) and Zo-1 (**C**) in the colon tissues from HCT116-engrafted mice with *Fn* colonization. (**D**) Immunoblot analysis of Claudin-1 in the colon tissues from different groups of mice engrafted with HCT116 cells. (**E**) The relative ratios of Claudin-1 to β-actin from D were quantitated. Data were expressed as mean ± SD, and *p* < 0.05 was considered statistically significant. * *p* < 0.05, ** *p* < 0.01, vs. *Fn* group. #### *p* < 0.0001, vs. Control.

Since SNH was administered by oral gavage and absorbed by the gut into systemic circulation, we further analyzed the potential adverse effect of SNH in vivo. The effects of SNH on the integrity of the intestinal barrier were tested by 4 kD FITC-Dextran permeability assay. SNH did not alter the permeability of the intestinal barrier at both low and high doses, while MET caused a mild disruption of the intestinal barrier (Figure 7A). To further confirm this, we measured the expression of key genes involved in the gut vascular barrier (GVB) and the intestinal epithelial barrier, *Claudin* and *Zo-1*. *Fn* reduced expression mRNAs encoding the tight junction proteins Claudin-1 and Zo-1 (Figure 7B,C). Whereas SNH rescued the expression of *Claudin-1* and *Zo-1* mRNAs (Figure 7B,C). Consistent with this, immunoblot data showed similar changes in the protein levels of Claudin (Figure 7D,E and Figure S4), indicating the improvement of the intestinal barrier induced by SNH. The results indicate that SNH has no adverse effect on the permeability of the intestinal barrier, and rescued the intestinal barrier damaged by *Fn*, a critical factor for anti-cancer treatment. Of note, although SNH and MET had similar anti-*Fn* effects, SNH showed no apparent side effects, as evidenced by decreased intestinal permeability.

### 3.6. SNH Inhibits Fn-Induced Inflammation

**Figure 8:**
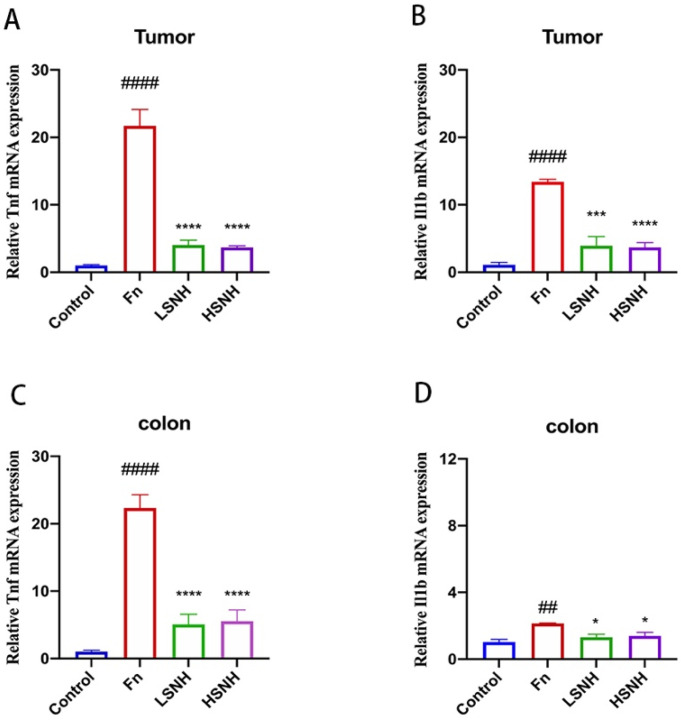
Alleviation of *Fn*-induced tumors growth and inflammatory response by SNH treatment. (**A**–**D**), qPCR analysis of the expression of genes encoding the proinflammatory cytokines TNF-α (**A**,**C**) and IL-1β (**B**,**D**) in the tumors and the colon tissues of the HCT116-engrafted mice with *Fn* colonization. All values were represented as mean ± SD, and *p* values < 0.05 were considered statistically significant. * *p* < 0.05, *** *p* < 0.001, **** *p* < 0.0001, vs. *Fn* group. ## *p* < 0.01, #### *p* < 0.0001, vs. Control.

Cancer progression is driven by both intrinsic oncogenic factors and extrinsic environmental cues, such as bacterial infection [36,37,38,39]. Bacteria drive tumor progression largely through cancer-driven inflammation, induced by bacteria and bacteria-induced inflammation, is closely associated with CRC progression [40,41]. To confirm the inhibition of *Fn* by SNH, we tested the effects of SNH on the *Fn*-induced inflammation in vivo, and the inflammatory cytokines TNF-α and IL-1β in the tumor xenografts and colon tissues from the HCT116-engrafted mice were analyzed by qPCR. *Fn* inoculation significantly increased the expression of genes encoding the proinflammatory cytokines TNF-α and IL-1β in the tumor xenografts and colon tissues (Figure 8A–D). Consistently, low-dose SNH or high-dose SNH markedly reduced the expression of TNF-α and IL-1β in both tumors and normal colon tissues (Figure 8A–D). The above results showed that SNH reduced *Fn*-associated tumor progression by directly inhibiting *Fn* and cancer-driven inflammation.

## 4. Discussion

*Fn*, one of the common anaerobes in the human oral cavity, was also found to be enriched in CRC tissues [9]. The enrichment of *Fn* in CRC tumors promotes colorectal carcinogenesis, cancer metastasis and chemoresistance [7,8]. In addition, *a* high abundance of *Fn* is associated with a poor prognosis in patients. However, current treatments of CRC, including surgery, chemotherapy, radiotherapy, targeted therapy, and immunotherapy, pay little or no attention to the serious impacts of *Fn* on CRC progression and there is no specific antimicrobial cancer therapy throughout the course of cancer treatment. In this study, we found a new antibacterial agent against *Fn* from herbal medicine screening, named SNH, and characterized its anti-tumor effects on CRC tumor growth and proliferation. SNH showed little cytotoxicity toward cancer cells and normal cells in vitro and in vivo. Mechanistically, SNH inhibits *Fn* through direct disruption of the bacterial outer membrane and inner membrane. More importantly, by inhibiting *Fn*, SNH reduced the *Fn*-associated CRC progression.

Herbal medicine has a rich and long history of discovering drugs with a remarkable curative effect and relatively low cost. Among the six common herbal chemicals, SNH, icariin, baicalin methyl ester, acteoside, salidroside, and echinacoside, SNH has the best antibacterial activity against *Fn* (Table 1 and Figure S1). SNH is a derivative of houttuynin, the main active ingredient of houttuynia cordata thumb [42]. Structurally, SNH is more stable than houttuynin [42]. Currently, SNH is an antibacterial agent mainly for inflammatory diseases, such as chronic bronchitis, pneumonia, and other respiratory diseases, which is effective for infections caused by *Staphylococcus aureus*, *Streptococcus pneumoniae, Streptococcus mutans, Pseudomonas aeruginosa* and *Candida albicans* [34,43,44,45,46]. MET, as a common clinical antibacterial agent against anaerobic bacteria, is widely used in the treatment of infectious diseases. Although SNH has a higher number of MIC and MBC in vitro, compared with MET, our data showed that SNH had a strong antibacterial effect on *Fn* in vitro and in vivo particularly, inhibiting the tumor-promoting effect of *Fn*. *Fn* promoted the CRC progression, but oral administrations of SNH reduced the tumor weight and tumor volume of xenograft tumors from CRC cells-engrafted mice with *Fn* colonization at a level similar to that of the mice without *Fn* (Figure 5). In addition, SNH effectively reduced *Fn* load in the mice xenograft tumors with *Fn* colonization.

Although MET may have better antibacterial activity against *Fn* than SNH in vitro, SNH and MET showed the same antibacterial effect in vivo. CRC is susceptible to bacteria and inflammation and CRC progression is driven by intrinsic oncogenic signaling and extracellular environmental cues, such as bacterial infection and cancer-driven inflammation. SNH might be used as a potential adjuvant treatment for CRC to overcome the current obstacles of first-line CRC drugs, including high recurrence rates and adverse side effects to normal tissues/cells. Our study of SNH has clinical significance. At present, SNH has been clinically available. Future studies are needed to identify the broader anti-microbiota spectrum, in particular both intracellular and extracellular bacteria that induce inflammation and drive CRC progression. By targeting bacteria, SNH-based treatment may provide a very promising adjuvant treatment for CRC.

Parenteral and enteral nutrition provide necessary nutrients for cancer patients. Chemotherapy drugs often damage intestinal mucosa, resulting in the disruption of nutrient absorption and intestinal homeostasis, as well as related immune functions [47,48]. These adverse effects may limit the treatment of CRC patients. Consistent with the previous reports [49,50], we found that MET causes gut damage and permeabilization of the intestinal barrier in CRC mice after 18 days of oral administration (Figure 7). However, at similar doses, SNH showed no damage to the intestinal mucosa and increased the expression of tight junction proteins. Our data indicate that SNH is a better anti-bacterial drug than MET, due to its cytotoxicity in vitro and in vivo. In addition, because of the widespread utilization of MET, increasing resistance to MET has become a problem that cannot be ignored [51]. SNH may provide an alternative to MET for the treatment of infectious diseases.

Regarding the mechanism of anti-bacterial activity of SNH, a previous study showed that SNH may induce the formation of H_2_O_2_ that contributes to the death of aerobic bacteria *Streptococcus pneumoniae* by proteomic analysis [34]. But bacteria located in the intestinal cavity and inside the tumors are most anaerobic. Direct effects of SNH on anaerobe have been little investigated. The results showed that SNH did not lead to the H_2_O_2_ formation of anaerobic *Fn* (Figure 1). Our study found that SNH disrupted cell membrane integrity which mediates its bactericidal effect. NPN can have access to the inner membrane by passing through the outer membrane of *Fn* with the help of SNH, thus exhibiting a strong fluorescent signal. Staining with membrane-impermeable DNA-specific dye PI further confirmed that SNH at the low concentration (<MIC) induced the accessibility of PI to the *Fn* genome. In addition, the segregated fluorescent signal of membrane colorant FM4–64 was significantly decreased after treatment with SNH. Our results showed that the integrity of the cell membrane of *Fn* was disrupted by SNH. Bacterial proteins FadA are major membrane proteins closely related to cell binding and pathogenicity of *Fn* [52]. Based on the molecular docking, they might be potential targets of SNH. FadA is closely associated with cell binding and enhancing pathogenicity [52]. SNH might effectively bind to FadA to disrupt the membrane integrity, thereby reducing the viability of *Fn* and the expression of mRNA, including FadA in the tumors and colon tissues of CRC mice xenografts with *Fn* colonization. Since FadA is a unique protein of Fusobacterium [53], it will be an ideal target to specifically inhibit *Fn*-mediated CRC and the target and mechanism of SNH are worthy of further investigation in the future.

## 5. Conclusions

In conclusion, our study showed that SNH had potent antibacterial activity on *Fn*, and it reduced the *Fn* load in tumor tissues, thus effectively inhibiting the tumor growth of mice xenografts with *Fn*. The antimicrobial activity of SNH was mediated by disrupting the membrane integrity of *Fn* and the *Fn-*associated cancer-driven inflammation. Meanwhile, the impaired intestinal barrier was improved by SNH. Our findings of SNH with potent antibacterial activity against *Fn* are relevant to the treatment regimens for patients with CRC. Since *Fn* is associated with the recurrence, metastasis and chemoresistance of CRC, the anti-*Fn*-based treatment should be considered for future treatment of CRC, in particular drugs such as SNH with little or no observable side effects at their effective anti-microbial doses. SNH could also be utilized for antimicrobial treatment, especially for patients with existing antibiotic allergies or resistances. Furthermore, our study of SNH has clinical significance. SNH has been clinically available, and it might be used as a potential adjuvant for CRC. In addition, antimicrobial therapy with SNH could be a good therapy for CRC prevention.

## Figures and Tables

**Table 1:** MIC and MBC of herbal chemicals against *Fusobacterium nucleatum (Fn)*.

Name	SNH	Icariin	Baicalin Methyl Ester	Acteoside	Salidroside	Echinacoside	Metronidazole
MIC	200 μM	>320 μM	>320 μM	>320 μM	>320 μM	>320 μM	0.125 μM
MBC	2000 μM	ND	ND	ND	ND	ND	0.5 μM

ND: Not Determined.

## Data Availability

The data and materials for this study are available by contacting the corresponding author upon reasonable request.

## Supplementary Materials

The following supporting information can be downloaded at: https://www.mdpi.com/article/10.3390/cancers14246111/s1. Figure S1: Structures of phytochemicals from herbal medicine. Figure S2: Antimicrobial activities of phytochemicals from herbal medicine against *Fn*. Figure S3: Immunoblot analysis of Caspase 3 and cleaved-Caspase 3 in HCT116-xenograft tumors. Figure S4. Full Immunoblot images. Table S1: The primer sequences for qPCR assay [54].
