# Supplementary material for: Sodium New Houttuyfonate Inhibits Cancer-Promoting Fusobacterium nucleatum (Fn) to Reduce Colorectal Cancer Progression
Source: Cancers (Basel). 2022 Dec 12;14(24):6111. doi: 10.3390/cancers14246111 (PMC9775898; doi:10.3390/cancers14246111)
Supplement: Supplementary file 1 [file cancers-14-06111-s001.zip › cancers-2075243-supplementary.pdf]

Supplemental Materials and Methods

1.1. Minimum inhibition concentration (MIC) assay

The minimum inhibitory concentration of herbal chemicals against *Fusobacterium nucleatum* (*Fn*, ATCC 25586) was determined by the broth microdilution method [1]. Briefly, *Fn* was cultured in brain heart infusion broth (BD Difco, USA). The concentration of bacterial inoculum was 3 × 10^8 CFU/ml. The control group was added with liquid medium without herbal chemicals. Metronidazole (MET) was used as a positive control. Each group was set up in triplicate and incubated for 24 h at 37 °C. The experiment was repeated at least three times to ensure reproducibility.

1.2. Minimum bactericidal concentration (MBC) assay

MBC is the minimum concentration of herbal chemicals required to kill bacteria. The mixture of the drug at various concentrations above the MIC and the bacteria solution was added to sterile agar plates and incubated at 37 ° C for 24 h to observe the number of colony forming units (CFU) [1]. The concentration that reduced the viability of *Fn* by ≥99.9 % was defined as the MBC of herbal monomers.

1.3. Detection of intracellular hydrogen peroxide

Intracellular hydrogen peroxide was assayed using Hydrogen Peroxide (H_2O_2) Content Assay Kit (BC3595, Solarbio, China). Briefly, bacterial inoculum (5x10^8 CFU/ml) was treated with different concentrations of SNH and centrifuged at 37 °C for 2h to collect the bacteria. Bacteria medium without SNH was a control. The cells were broken up by ultrasound (ultrasonic 3s, 10s, repeat 30 times) and the supernatant was collected after centrifugation (8000 rpm) at 4°C for 10 min to measure the concentration of hydrogen peroxide (H_2O_2) according to the manufacturer's protocol.

1.4. Outer membrane permeability of Fn

The effect of SNH on the outer membrane permeability of *Fn* was determined by using hydrophobic fluorescent probe N-Phenyl-1-naphthylamine (NPN, Aladdin, Shanghai, China) as previously described [1]. Briefly, after PBS washing, *Fn* was resuspended in PBS and adjusted to an optical density (OD_{600nm}) of 0.5±0.02. SNH was used at 0.25-4 MIC in the SNH group (100 µl bacterial solution + 50 µl SNH+ 50 µl NPN (40 µM)) and PBS was used in the control group (100 µl bacterial solution + 50 µl PBS+ 50 µl NPN (40 µM)). NPN uptake was kinetically monitored for 13 minutes by multifunctional microplate reader at the excitation/emission wavelength of 350/420 nm.

1.5. Flow Cytometry Analysis

Fn was washed and suspended in PBS to an OD_{600nm} of 0.4. The diluted *Fn* solution was incubated with SNH at the concentrations ranged from 100 µM to 800 µM at 37 °C for 5 min. The *Fn*-SNH mixture was then washed and resuspended in PBS, followed by staining with Propidium Iodide (PI, 1 µg/ml) for 15 min [2]. The effect of SNH on the PI uptake by *Fn* was detected by flow cytometer (Becton Dickinson, USA).

1.6. Laser scanning confocal microscopy

Fn was washed and resuspended in PBS to an OD_{600nm} of 0.4, followed by incubation with SNH at the concentrations from 100 µM to 800 µM at 37 °C for 1 h. The *Fn*-SNH mixture was washed and resuspended in two groups: (1) PBS with PI (1 µg/ml) + Acridine Orange (AO, 1 µg/ml) to stain nucleic acid; (2) PBS with FM4-64 (1 µg/ml) + AO (1 µg/ml) to stain the membrane and nucleic acid. Microscopic analysis of membrane permeability of each dye was observed by laser scanning confocal microscopy (Leica TCS SP8).

1.7. Binding mode prediction

The binding model of SNH with *Fn* was predicted as previously described[3]. The crystal structures of key bacterial proteins FadA (PDB: 3ETW) were downloaded from the Protein Data Bank. Molecular

docking was carried out by Vina docking software, and the interaction between ligand and receptor was analyzed by PyMOL software to obtain binding energy, hydrogen bond, and other information.

1.8. Quantification of bacteria

RNA extraction, reverse transcription, and quantitative polymerase chain reaction (qPCR) were used to quantify *Fn* and gene expression as described previously [4, 5].

(1) RNA extraction:

Total RNA was extracted from CRC mouse xenografts using Trizol reagent (Beyotime, Shanghai, China) according to the manufacturer's instructions. In brief, 100 mg tumor tissues were added to 1 ml Trizol reagent and homogenized for 5 min at room temperature, followed by addition of 200 μl chloroform for phase separation. The aqueous phase containing RNAs was collected by centrifugation at 12,000 rpm at 4 °C for 10 min. Then, the equal volume of isopropanol was added to the aqueous phase, mixed, and incubated at room temperature for 10 minutes before centrifugation at 12,000 rpm for 10 min at 4 °C to collect the RNA pellet. 500 μl rinse solution was added and incubated at room temperature for 2 min before the solution was added to a 1.5 ml tube with the RNA-binding column for centrifuged at 12,000 rpm for 2 min at 4 °C. 30 μl RNase free ddH_2O was used to elute RNA from the column by centrifugation at 12,000 rpm at 4 °C for 2 min.

(2) Reverse transcription:

3 μl 5×gDNA Digester Mix (Yeasen, Shanghai, China) was added to the 0.2 ml PCR tubes, followed by addition of total RNA and ddH_2O in a reaction system of 15 μl. The mixture was heated at 42 °C for 2 min to remove genomic DNA, and then cooled down in ice. After quick spin-down, 5 μl 4×Hifair®IIISuperMix Plus (Yeasen, Shanghai, China) was added to the above mixture for reverse transcription at 55 °C for 15 min.

(3) qPCR

qPCR was performed with Hieff® qPCR SYBR Green Master Mix (Yeasen, Shanghai, China) according to the manufacturer's instructions. Briefly, 3.6 μl ddH2O, 0.2 μl primers (10 μM), 1μl cDNA templates, and 5 μl Hieff® qPCR SYBR Green Master Mix were added sequentially into 96-well plates. Reaction program was as follows: 10 min at 95 °C, and 45 cycles of 15 s at 95 °C, and 1 min at 60 °C by StepOne Plus real-time PCR system (Applied Biosystems, USA). Relative mRNA expression was calculated from the comparative cycle threshold (CT) values relative to that of reference gene GAPDH. Data are presented as arbitrary units and were calculated by 2^(–ΔΔ CT) method. Sequences of primers are listed in Table S1.

1.9. Assessment of intestinal permeability

After overnight fasting, mice were intragastrically gavaged with 4 kD FITC-Dextran (0.4 mg/g body weight) and 4 h later, the mice were anesthetized by intraperitoneal (i.p.) injection of pentobarbital sodium to collect blood from the retro-orbital sinus into EDTA-treated 1.5 ml tubes. 200 μl plasma was collected by centrifugation (3500 rpm) for 10min at 4°C and diluted in PBS at a ratio of 1:4 to add to a 96-well black plate for FITC-Dextran concentration measurement using a multifunctional microplate reader at the excitation/emission wavelength of 480/520 nm. The effect of SNH on the expression of tight junction proteins Claudin-1 and Zonula occludens protein 1 (ZO-1) were analyzed by qPCR to quantify tight junction proteins. Western blot was performed as described previously[6]. Antibodies were used in the present study: β-actin (3700S, CST, 1:1000 dilution), Claudin-1(ab242370, abcam, 1:5000 dilution) and Caspase-3 (ab184787, abcam, 1:1000 dilution). The images were analyzed by ImageJ software.

1.10. Assessment of the expression of the proinflammatory cytokines

The effect of SNH on the expression of the proinflammatory cytokines tumor necrosis factor α (TNF-α) and interleukin-1β (IL-1β) in HCT116-engrafted mice with *Fn* inoculation were detected by qPCR. After the last intragastric administration of SNH or control, mice with HCT116-xenograft and *Fn* inoculation were sacrificed for the collection of xenograft tumors and colon tissues. RNA extraction, reverse transcription, and qPCR were used to quantify proinflammatory cytokines as described previously.

Table S1. The primer sequences for qPCR assay.

Name	Primer sequences (forward primer)	Primer sequences (reverse primer)
16S	5’-GGTGAATACGTTCCCGG-3’	5’-TACGGCTACCTTGTTACGACTT-3’
Fn	5’-CAACCATTACTTTAACTCTACCATGTTCA-3’	5’-GTTGACTTTACAGAAGGAGATTATGTAAAAATC-3’
FadA	5′-TGATGCAGCAAGTTTAGTAGGTGAA-3′	5′-TGCTAGTGCTTGTCTAGCAGCG-3′
Claudin	5’-GTCTTTGACTCCTTGCTGAATCTG-3’	5’-CACCTCATCGTCTTCCAAGCAC-3’
Zo-1	5’-GGGAGGGTCAAATGAAGACA-3’	5’-GGCATTCCTGCTGGTTACAT-3’
Tnf	5’-AGGGTCTGGGCCATAGAACT-3’	5’-CCACCACGCTCTTCTGTCTAC-3’
Il1b	5’-GGTCAAAGGTTTGGAAGCAG-3’	5’-TGTGAAATGCCACCTTTTGA-3’

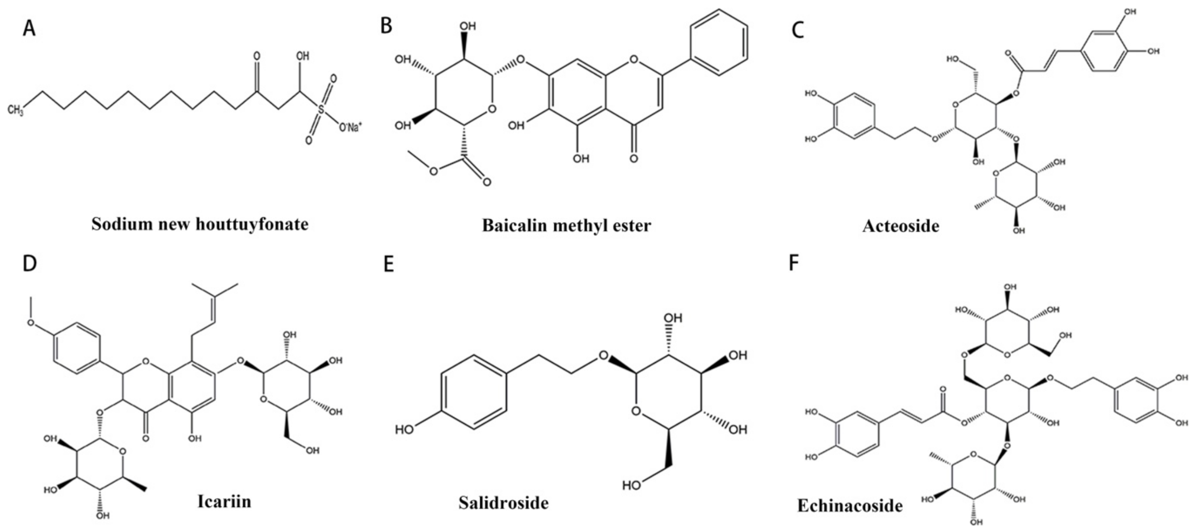

Figure S1. Structures of phytochemicals from herbal medicine. Structures of SNH (A), Baicalin methyl ester (B), Acteoside (C), Icariin (D), Salidroside (E), and Echinacoside (F).

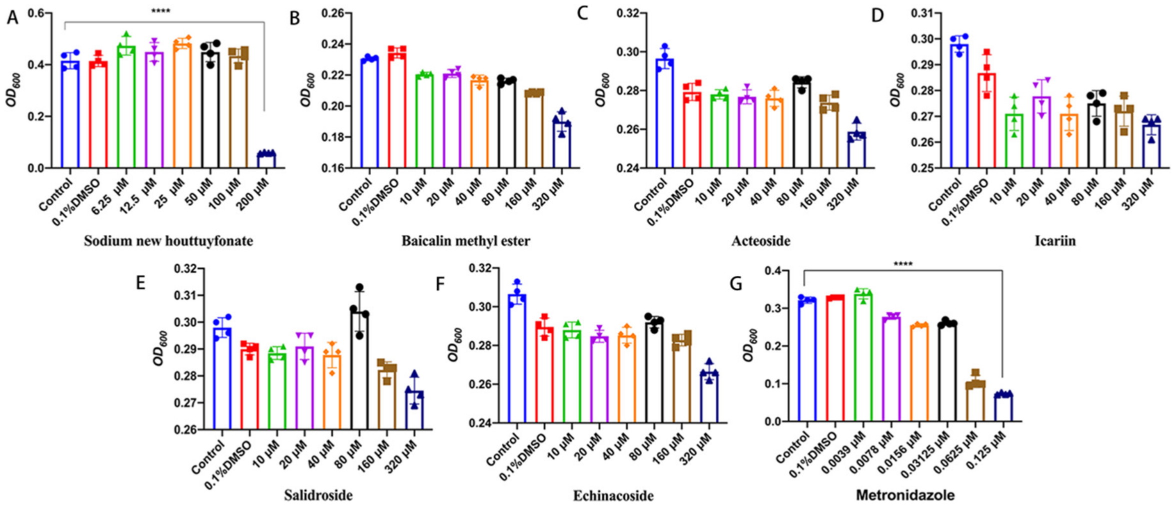

Figure S2. Antimicrobial activities of phytochemicals from herbal medicine against *Fn*. Effects of Sodium new houttuyfonate (A), Baicalin methyl ester (B), Acteoside (C), Icariin (D), Salidroside (E), Echinacoside (F), and Metronidazole (G) on the viability of *Fn.* Each experiment was repeated three times independently.

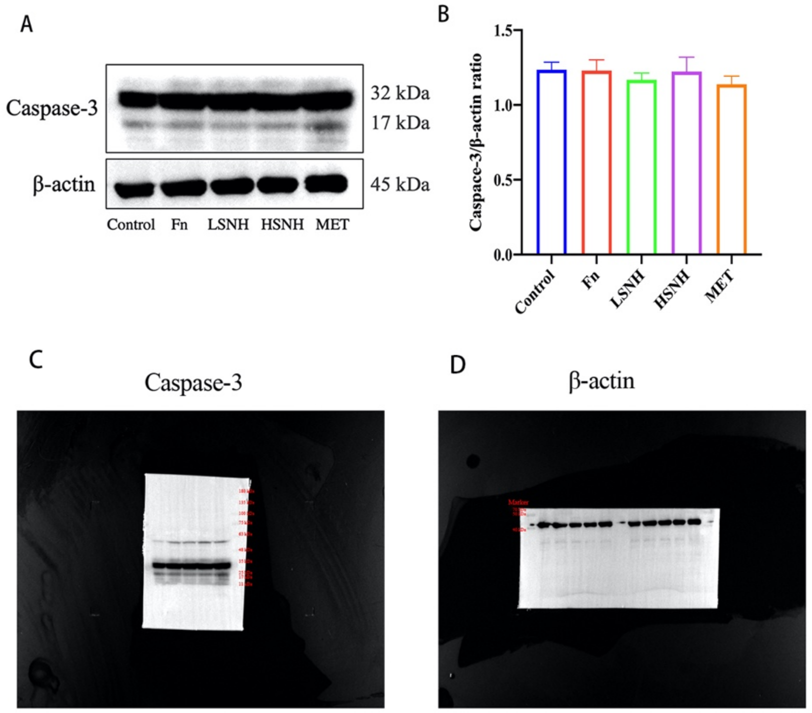

Figure S3. Caspase 3 was detected in HCT116-xenograft tumors. (A) Western blot was performed in different groups of HCT116-xenograft tumors. (B), Quantitation of Caspase-3/β-actin expression in A. (C,D) Full Immunoblot images of Caspase-3(C), and β-actin (D).

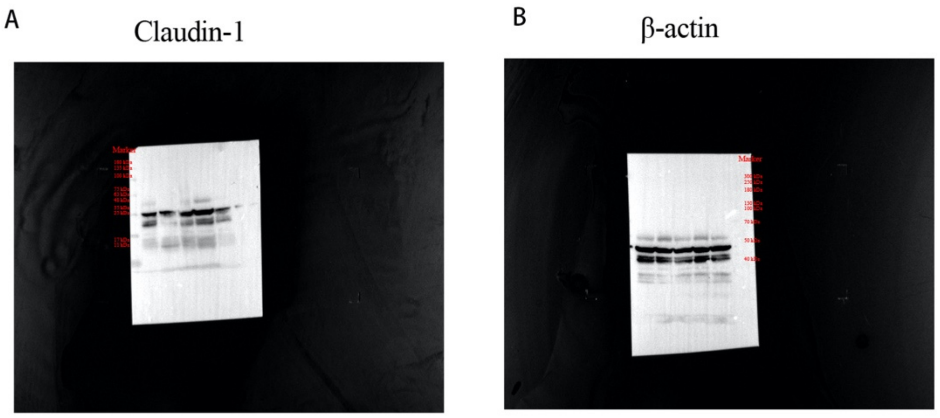

Figure S4. Full Immunoblot images of Claudin-1(A) and β-actin (B) as shown in Figure 7D.

References

1. Jia F, Zhang Y, Wang J, Peng J, Zhao P, Zhang L, Yao H, Ni J, Wang K. The effect of halogenation on the antimicrobial activity, antibiofilm activity, cytotoxicity and proteolytic stability of the antimicrobial peptide Jelleine-I. *Peptides.* **2019**, *112*, 56-66.
2. Jia F, Liang X, Wang J, Zhang L, Zhou J, He Y, Zhang F, Yan W, Wang K. Tryptic Stability and Antimicrobial Activity of the Derivatives of Polybia-CP with Fine-Tuning Modification in the Side Chain of Lysine. *International Journal of Peptide Research and Therapeutics.* **2021**, *27*, 851-862.
3. Ferreira LG, Dos Santos RN, Oliva G, Andricopulo AD. Molecular docking and structure-based drug design strategies. *Molecules.* **2015**, *20*, 13384-13421.
4. Yu T, Guo F, Yu Y, Sun T, Ma D, Han J, Qian Y, Kryczek I, Sun D, Nagarsheth N *et al.* Fusobacterium nucleatum Promotes Chemoresistance to Colorectal Cancer by Modulating Autophagy. *Cell.* **2017**, *170*, 548-563.e516.
5. He F, Antonucci L, Yamachika S, Zhang Z, Taniguchi K, Umemura A, Hatzivassiliou G, Roose-Girma M, Reina-Campos M, Duran A *et al.* NRF2 activates growth factor genes and downstream AKT signaling to induce mouse and human hepatomegaly. *J Hepatol.* **2020**, *72*, 1182-1195.
6. Xiong H, Hong J, Du W, Lin Y-w, Ren L-l, Wang Y-c, Su W-y, Wang J-l, Cui Y, Wang Z-h *et al.* Roles of STAT3 and ZEB1 Proteins in E-cadherin Down-regulation and Human Colorectal Cancer Epithelial-Mesenchymal Transition*. *Journal of Biological Chemistry.* **2012**, *287*, 5819-5832.
